# Adenosine-producing regulatory B cells in head and neck cancer

**DOI:** 10.1007/s00262-020-02535-6

**Published:** 2020-03-07

**Authors:** Sandra S. Jeske, Matthias Brand, Andreas Ziebart, Simon Laban, Johannes Doescher, Jens Greve, Edwin K. Jackson, Thomas K. Hoffmann, Cornelia Brunner, Patrick J. Schuler

**Affiliations:** 1 Department of Oto-Rhino-Laryngology, Head and Neck Surgery, Ulm University Medical Center, Frauensteige 12, 89075 Ulm, Germany; 2 Department of Neurosurgery, Mannheim University Medical Center, Mannheim, Germany; 3 Department of Pharmacology and Chemical Biology, University of Pittsburgh School of Medicine, Pittsburgh, PA USA

**Keywords:** Regulatory B cells, Adenosine, BTK, Head and neck cancer, ADORA_2A_

## Abstract

**Background:**

Multiple mechanisms of immunosuppression have been identified in the tumor microenvironment including regulatory B cells (B_reg_). Recently, we have shown that B_reg_ suppress T cell function by production of adenosine (ADO). However, the autocrine effect of ADO on B cells and the role of B_reg_ in head and neck cancer remains unclear.

**Methods:**

Blood (*n* = 42) and tumor tissue (*n* = 39) of head and neck cancer patients and healthy donors (*n* = 60) were analyzed by FACS. The effect of ADO on phenotype, intracellular signaling pathways, Ca^2+^ influx and ADO production was analyzed in B_reg_ and effector B cells (B_eff_) by FACS, luminescence and mass spectrometry. The blockage of the ADO receptor A_2A_ was analyzed in a murine head and neck cancer model.

**Results:**

ADO-producing B_reg_ were found in tumor tissue and peripheral blood. ADO inhibited the intracellular Bruton’s tyrosine kinase (BTK) and Ca^2+^ influx only in B_eff_. The inhibition of BTK by ibrutinib mimicked the effect of ADO, and ibrutinib reduced the production of ADO by downregulation of CD39 in vitro. The inhibition of ADO receptor A_2A_ significantly reduced tumor mass and increased B cell infiltration, in vivo.

**Conclusion:**

Our data demonstrate the presence of a novel ADO-producing B_reg_ population within the tumor microenvironment in mice and humans. A new model is proposed on how ADO-producing B_reg_ can influence the function of B_eff_ cells in healthy donors and cancer patients. Thus, the modulation of the ADO pathway in B cells may serve as a therapeutic approach for cancer patients.

**Electronic supplementary material:**

The online version of this article (10.1007/s00262-020-02535-6) contains supplementary material, which is available to authorized users.

## Introduction

Head and neck squamous cell carcinoma (HNSCC) belongs to the most aggressive cancers worldwide, with a 5-year survival rate less than 50% for advanced cancers [1]. As compared to other tumor entities, HNSCC is regarded as an immunogenic tumor likely to be responsive to immunotherapeutic approaches [2]. Therefore, the approval of the checkpoint inhibitor nivolumab in 2017 has attracted particular attention [3]. However, current immunotherapeutic efforts are mostly focused on T_helper_, T_killer_ and regulatory T cell (T_reg_) populations.

Although B cells are a part of the adaptive immune system, they have received little attention in oncological immunotherapy of solid tumors. Recent discoveries demonstrate that B cells have a decisive influence on solid tumor growth and thus can also serve as a therapeutic target. It is now clear that, in addition to antibody production, B cells can also process antigens or acquire immunosuppressive properties. Accordingly, a detailed analysis of defined B-cell populations and their contribution to cancerogenesis is needed.

Until today, it remains unclear whether B cells have pro- or anti-tumorigenic features. In a variety of murine tumor models, B cells have been shown to suppress T cells and increase tumor growth [4, 5]. On the other hand, tumor-infiltrating B cells are associated with a good outcome in patients with lung, breast, ovary or head and neck cancer [6–9]. In addition, lymphoid structures containing T and B cells have been recently discovered in solid tumor tissue [10]. These structures enable tumor antigen contact, which may support the endogenous anti-tumor immunity [11]. Possibly, these conflicting results on the role of B cells in the tumor environment are due to different B cell populations influencing tumor growth in opposing ways. For example, there are many subsets of regulatory B cell populations (B_reg_), which are defined by their phenotype and function. Previously, we have described a population of B_reg_ in healthy donors, which express the ectonucleotidases CD39 and CD73 on their surface and produce immunosuppressive adenosine (ADO) [12]. In the peripheral blood of healthy donors, almost 70% of all CD19^+^ B cells carry both enzymes on their surface.

The importance of ADO in the tumor environment has been known in the past. Solid tumors contain high levels of exogenous immune-suppressive ADO [13, 14], and the inhibition of the adenosine receptor A_2B_ (ADORA_2B_) signaling leads to a decreased proliferation of human HNSCC cells, in vitro [15]. HNSCC patients with low levels of ADORA_2A_ have a significantly better overall survival rate which correlates with low levels of T_reg_ and a high level of CD8^+^ T cells [16]. In addition, both a selective ADORA_2A_ inhibitor and a CD73 antibody are currently tested in clinical phase I studies for solid tumors in combination with a PD-1 antibody (NCT02403193/NCT02503774).

The following study reports on the presence of a novel ADO-producing B_reg_ population within the tumor microenvironment (TME) in mice and humans. A new model is proposed on how ADO-producing B_reg_ can influence the function of B_eff_ cells in healthy donors and cancer patients. The discussion includes the modulation of the ADO pathway in B cells as a therapeutic approach for cancer patients.

## Materials and methods

### Human blood samples

Whole blood samples were obtained from tumor patients (*n* = 42) and healthy volunteers (*n* = 60). Peripheral blood mononuclear cells (PBMC) were isolated by Biocoll Separating Solution (Merck, USA) or Pancoll (density 1119 g/ml and 1077 g/ml; PAN Biotech, Germany) and directly used for experiments. Clinicopathological and demographic parameters are listed in Supplementary table S1.

### Tumor tissues processing

Human tumor tissue (*n* = 39) was collected during surgery in NaCl. Minced tissue pieces were collected in RPMI containing 200 IU/ml collagenase I (Worthington, USA) for at least two hours at 37 °C and mashed with a 100 µm EASY strainer (Gibco, USA). The lymphocyte fraction was isolated by Biocoll centrifugation. The purity of the cells was measured by anti-CD45 staining.

### FACS antibodies

Antihuman: p-BTK (pY223) PE, CD19 PE-Cy5, IgM PE, CD45 AmCyan and CD45 FITC (Becton Dickinson, USA), CD39 PE-Cy7, CD73 FITC, CD73 eFluor450, CD21 FITC and CD23 APC (eBioscience, USA). Anti-mouse: B220 FITC, CD4 PerCP, CD45 APC-Cy7 (BD-Pharmingen, USA), IgM PE (Southern Biotech, USA) and CD3e eFlour450, CD39 PE-Cy7, IgD FITC, IgM APC (eBioscience, USA), IgD APC-Cy7, CD73 PacificBlue, CD19 PE-Cy7 and CD8a PE (BioLegend, USA). All human mAbs were titrated using normal PBMC to establish optimal dilution.

### Surface staining

Cells were stained as previously published [17]. All flow cytometry measurements were taken using a Gallios 10-color flow-cytometer equipped with Kaluza flow cytometry software (both Beckman Coulter, USA). At least 10^5^ cells were acquired for analysis.

### Cell separation

CD19^+^ human B cells were separated from PBMC using the Human B cell Enrichment Kit or the Human B cell Isolation Kit (both stemcell, Canada) according to the manufacturer’s instructions. The purity of the separated cells as monitored by flow cytometry was always ≥ 93%. CD19^+^ mouse B cells were separated from spleen cells using the Mouse CD19 Positive Selection Kit II, and CD4^+^ and CD8^+^ T cells were separated after PE staining with the PE Selection Kit (both stemcell, Canada) according to the manufacturer’s instructions.

### B-cell culture

B cells were cultured in 96-well plates (2.5 × 10^4^ cells/well) containing RPMI + 10% FBS superior (Merck, USA), 100 U/ml penicillin and 100 µg/ml streptomycin (Pan Biotech, Germany). B cells were stimulated with IL-4 (1,000 IU/ml, CellGenix, Germany), CD40L (2 µg/ml) and hemagglutinin (423 ng/ml) (both R&D, Canada) and cultured for 6 days (37 °C, 5% CO_2_). In addition, some cells were treated with ibrutinib (10 µM, Pharmacyclics, USA). FACS analysis of surface markers (CD19, CD39, CD73) was performed before and after stimulation.

### Intracellular p-BTK staining

PBMC or isolated CD19^+^ B cells from healthy volunteers were stimulated with anti-µ-F(ab’)_2_ (5 µg/ml, Dianova, Germany). Some cells were incubated with ADO (1 mM) (Sigma-Aldrich, USA) for two hours or surface-stained with CD73 eFluor450 before stimulation. The stimulation was stopped by adding 10% formaldehyde solution (AppliChem, Germany). Cells were permeabilized with 0.1% Triton X-100 (Sigma-Aldrich, USA) kept in 50% methanol at − 20 °C for 10 min. Cells were washed and stained with p-BTK (pY223) PE for 45 min. at room temperature before FACS analysis.

### Calcium assay

Calcium assays were performed as described previously [18]. For stimulation, anti-µ-F(ab’)_2_ (5 µg/ml) was added immediately after baseline measurement. The addition of ionomycin was used as a positive control. Calcium flux was measured with the LSR Fortessa using the 355-nm UV laser (Becton Dickinson, USA).

### RT-PCR for ADO receptors

RNA was extracted and used for cDNA synthesis as described earlier [19]. Relative quantification of the target gene messenger RNA (mRNA) expression was calculated with the relative quantification in RT-PCR as previously published [20]. The expression levels of target genes were normalized to the levels of six endogenous controls (housekeeping genes RPL13A, β-actin, PBG-D, G6PD, GAPDH and TBP). The target genes of mouse cells were normalized to two housekeeping genes (GAPDH and actin). PCR primers and the respective UPL probes are listed in Supplementary table S2.

### ATP hydrolysis by luminescence

ATP measurements in the supernatants of B cells were taken with the ATPlite kit (PerkinElmer, USA) according to the manufacturer’s instructions and as published before [12]. All experiments were performed in technical triplicates.

### Mass spectrometry

Supernatants of B cells were centrifuged and boiled for 2 min at 95 °C to inactivate ADO-degrading enzymes and subsequently stored at − 80 °C for subsequent analysis as described before [12]. Purines were measured using liquid chromatography–tandem mass spectrometer by selected reaction monitoring with ^13^C_10_ ADO as the internal standard [12, 17]. The experiments were performed in triplicates.

### Animal model

Murine squamous carcinoma cells (SCC VII, 5 × 10^5^) were injected into the floor of the mouth of syngeneic C3H/HeJ mice (*n* = 72 including control groups) as described earlier [21, 22]. The experiments were started with 7–9-week-old male mice. Before tumor induction and 21 days after tumor injection, murine blood was analyzed by an animal blood counter (Scil animal care company GmbH, Germany) and by flow cytometry. On day 21, the tumor was resected and weighed. Tumor-infiltrating lymphocytes (TIL) were isolated and analyzed by flow cytometry as described above. For ADORA_2A_ inhibition, the ADORA_2A_ antagonist SCH-58261 (1 μg/g body weight in PBS; Tocris Bioscience, Great Britain) was injected intraperitoneally starting at day 7 after tumor induction and subsequently every fourth day [23].

### Statistical analysis

All data are presented as means of at least three experiments ± SD or as medians with interquartile range. Data were analyzed for Gaussian distribution with Shapiro–Wilk test. One-way and two-way ANOVAs were used for analyzing more than two values for statistical significance. The Wilcoxon matched samples test was used for nonparametric analysis of two mean values. *P* values ≤ 0.05 were considered to be significant. Statistical analyses were performed using Excel and GraphPad Prism version 6.01. Error bars indicate the SD of the mean data from replicate experiments.

## Results

### B_reg_ in human HNSCC patients

**Fig. 1:**
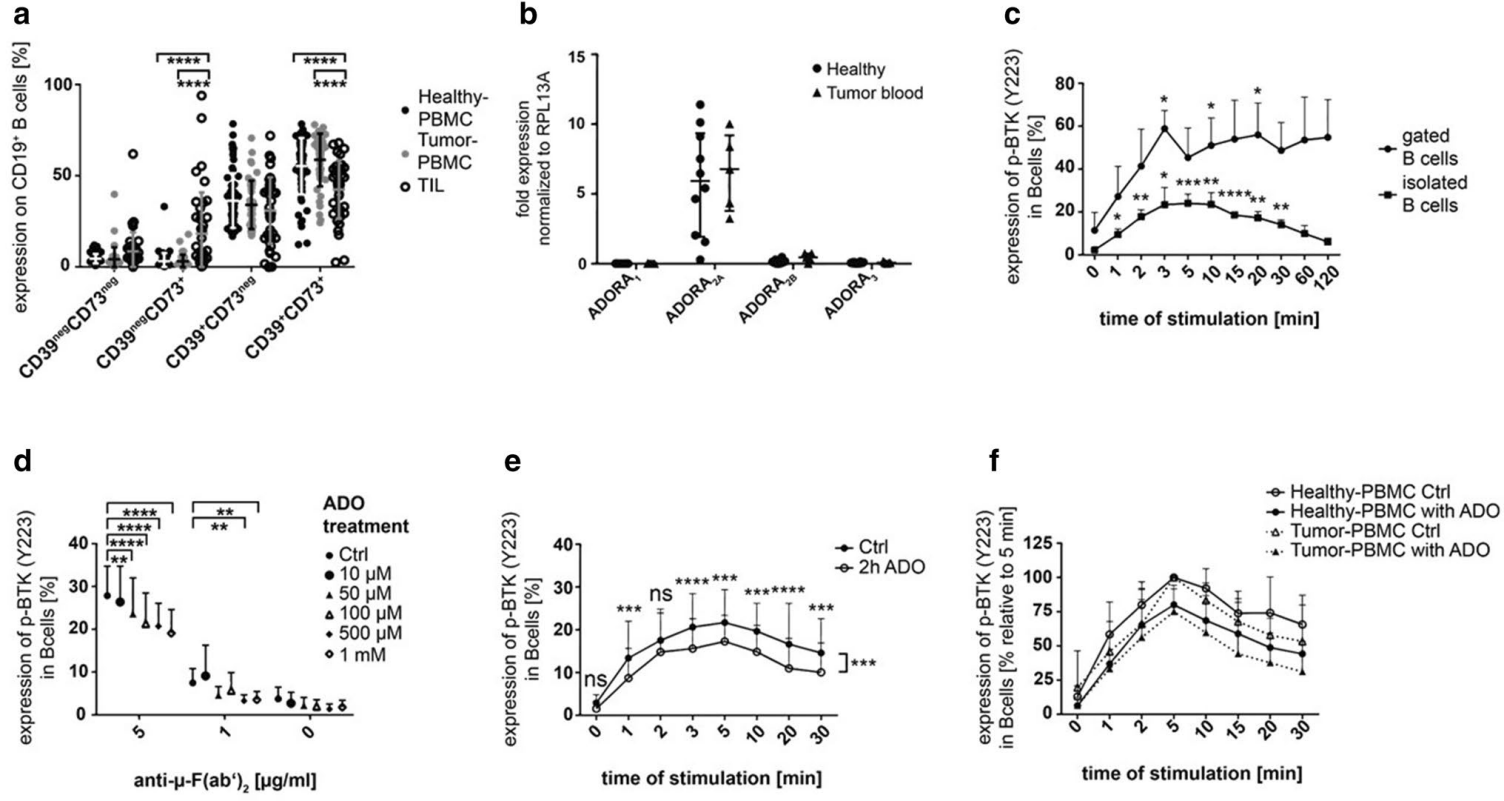
Human CD19^+^ B cells and their BTK phosphorylation in response to ADO. **a** The expression of the ectonucleotidases CD39 and CD73 measured by FACS in PBMC of healthy donors (*n* = 60), PBMC of tumor patients (*n* = 42) and in TIL of HNSCC patients (*n* = 39). Data are shown as means ± SD. **b** RNA levels for the four adenosine receptors (ADOR) in isolated peripheral B cells of healthy donors (*n* = 10) and tumor patients (*n* = 5). **c** Human B cells were stimulated with anti-µ-F(ab’)_2_ and stained for anti-p-BTK (pY223). The experiment was performed with gated B cells (*n* = 3) and isolated B cells in PBMC (*n* = 5). **d** Pre-incubation with ADO induced a decreased BTK phosphorylation in a concentration-dependent manner after stimulation with anti-µ-F(ab’)_2_ (*n* = 3). **e** Incubation with ADO decreased the ability of B cells to phosphorylate BTK in a time-dependent manner. Results are expressed as percentage of phosphorylated BTK shown as mean ± SD (*n* = 10). **f** The ability of ADO to decrease BTK phosphorylation in peripheral B cells is comparable in B cells of healthy donors (*n* = 10) and HNSCC patients (*n* = 7). Results are expressed as percentage of phosphorylated BTK. The five-minute value was set to 100%. All data are shown as mean ± SD. not significant (ns); *p* < 0.05 (*); *p* < 0.01 (**); *p* < 0.001 (***); *p* < 0.0001 (****)

ADO-producing B_reg_ are defined by the surface expression of the ectonucleotidases, CD39 and CD73. These B_reg_ were found in the peripheral blood of healthy donors (55.4 ± 15.5% of CD19^+^ B cells, *n* = 60) and tumor patients (58.8 ± 14.5%, *n* = 42). In the tumor microenvironment, the frequency of ADO-producing B_reg_ was significantly decreased as compared to the peripheral blood (42.6 ± 16%; *p* ≤ 0.0001, Fig. 1a). The CD39^+^CD73^+^ B cells in the peripheral blood and in the TME are mature follicular B cells (CD23^+^CD21^neg^).

### Adenosine receptors on B cells

We next aimed to describe the autocrine effect of ADO on B cells. Human B cells express mostly ADORA_2A_ with comparable expression levels in healthy volunteers and tumor patients (Fig. 1b). ADORA_2B_ and ADORA_3_ showed very low expression levels, while ADORA_1_ was almost undetectable in human samples.

### BTK phosphorylation in B cells

As phosphorylation of the Bruton’s tyrosine kinase (BTK) is a hallmark of B cell activation, we analyzed BTK phosphorylation (pY223) in B cells by FACS. Stimulation with anti-µ-F(ab’)_2_ induced phosphorylation of BTK in isolated CD19^+^ B cells as well as in PBMC gated on CD19^+^ B cells already after 1 min (Fig. 1c). However, gated B cells in PBMC showed a higher variance of BTK phosphorylation. We, therefore, decided to use isolated B cells for further experiments. Treatment of isolated B cells with different ADO concentrations prior to stimulation with anti-µ-F(ab’)_2_ induced a significant concentration-dependent reduction in the BTK phosphorylation (*p* ≤ 0.01; Fig. 1d). The effect of ADO was most pronounced, when cells were stimulated with a concentration of 5 µg/mL anti-µ-F(ab’)_2_, which was applied for further experiments. The inhibition of BTK phosphorylation in B cells by ADO (1 mM) lasted for at least 30 min (Fig. 1e). Peripheral B cells of tumor patients showed a similar response to ADO as compared with B cells of healthy volunteers (*p* = 0.09, Fig. 1f). These experiments demonstrate the ability of ADO to decrease BTK phosphorylation, which is a key molecule in the B-cell receptor (BCR)-mediated signaling pathway in the B cells of healthy donors as well as HNSCC patients.

### ADO decreases BTK phosphorylation exclusively in CD73^neg^ B cells

**Fig. 2:**
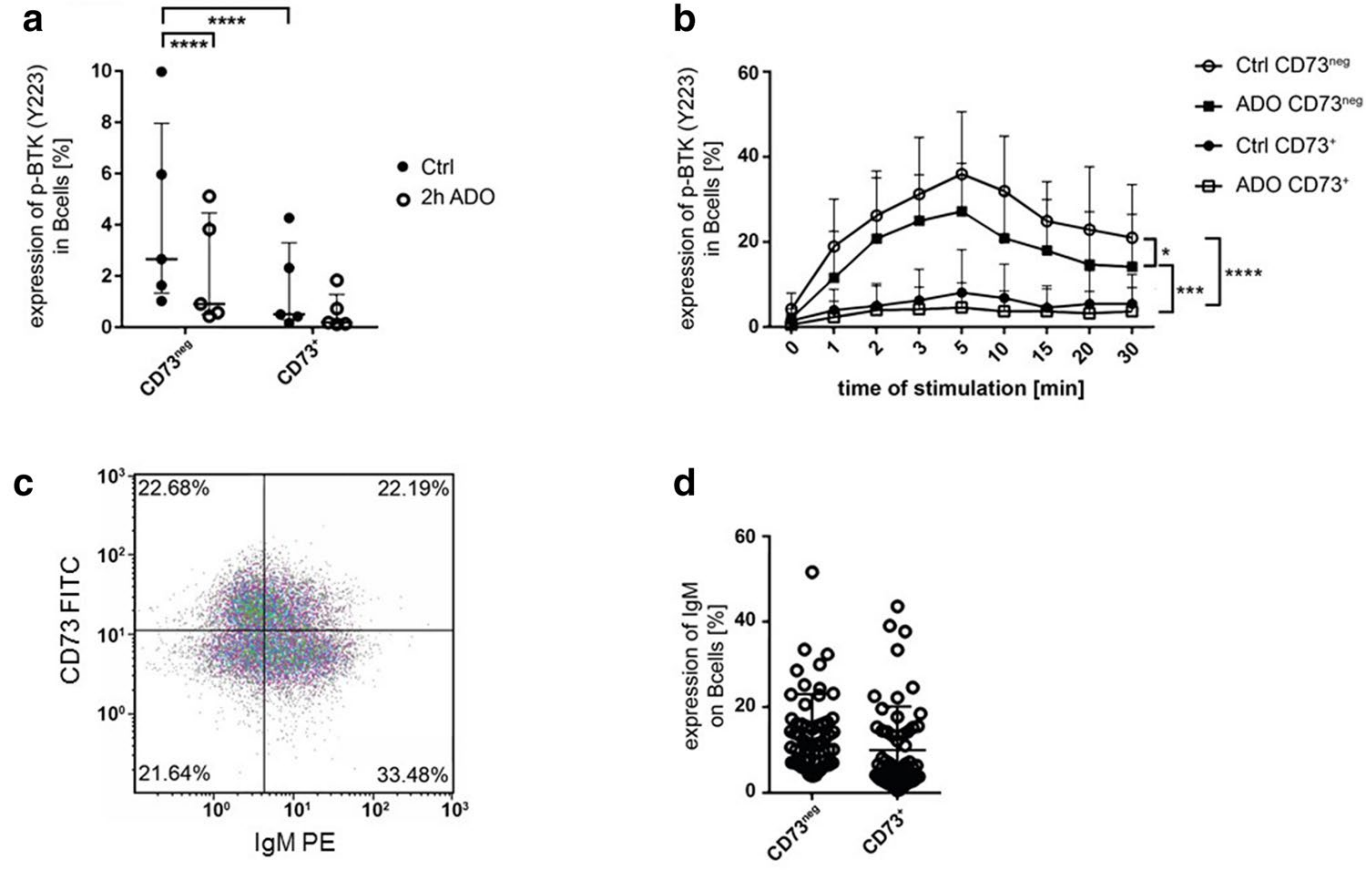
Phosphorylation of BTK in B cell subsets. **a** Unstimulated CD73^neg^ B_eff_ cells showed a significantly higher phosphorylation of BTK than CD73^+^ B_reg_ two hours after isolation and staining. After pre-treatment with ADO for 2 h, BTK phosphorylation was reduced in CD73neg B_eff_ cells. Data shown as median with interquartile range; paired t-test; *n* = 5. **b** Isolated human B cells were first stained for CD73 and then incubated with ADO for two hours. Phosphorylation was measured after stimulation with anti-µ-F(ab’)_2_. Incubation with ADO reduced BTK phosphorylation in CD73^neg^ B_eff_ cells. Analyzed with a two-way ANOVA; *n* = 5. **c** Expression of IgM and CD73 on CD19^+^ B cells in the density plot of one representative patient. **d** Frequency of IgM^+^ cells in the CD73^neg^ B_eff_ and CD73^+^ B_reg_ subset

Next, we investigated whether ADO-mediated downregulation of BTK phosphorylation was restricted to a specific subset of B cells. To this end, B cells obtained from healthy donors were stained with anti-CD73 in order to distinguish CD73^+^ ADO-producing B_reg_ from CD73^neg^ B_eff_ (Supplementary fig. S1a). In unstimulated B_reg_ as well as B_eff_, the level of p-BTK was low. Interestingly, basal p-BTK was significantly higher in unstimulated B_eff_ as compared to unstimulated B_reg_, and ADO treatment significantly decreased basal p-BTK only in unstimulated B_eff_, but not in unstimulated B_reg_ (Fig. 2a). Accordingly, after stimulation with anti-µ-F(ab’)_2_, p-BTK was only induced in B_eff_, but not in B_reg_, and the effect of stimulation was attenuated by ADO treatment (Fig. 2b). As anti-µ-F(ab’)_2_ specifically stimulates the µ-chain of the BCR IgM, we examined the expression of IgM on CD73^+^ B_reg_ and CD73^neg^ B_eff_ cells. Both B cell subtypes expressed comparable IgM levels on their surface and were therefore able to receive the stimulatory BCR signal (Fig. 2c, d). These observations suggest that BTK in CD73^+^ B_reg_ is less responsive than in CD73^neg^ B_eff_ cells.

### ADO decreases the ability of B cells to secrete Ca2+

**Fig. 3:**
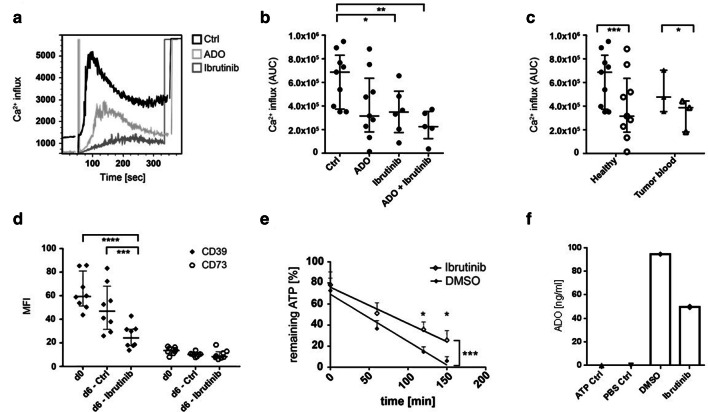
Calcium influx and B cell responses to treatment with the BTK inhibitor ibrutinib. **a** Isolated B cells were treated overnight with ADO and/or ibrutinib, stained with Indo-1 and stimulated with anti-µ-F(ab’)_2_ for direct Ca^2+^ measurement. The overlay plot of the kinetics shows control B cells in black, ADO-treated B cells in light gray and ibrutinib-treated B cells in dark gray of one representative experiment. The results were calculated by the following ratio: intensity indo-1 violet/indo-1 blue. **b** The Ca^2+^ influx was significantly reduced by ibrutinib and by the combination of ibrutinib and ADO. AUC = area under the curve as mean ± SD analyzed with one-way ANOVA; median with interquartile range; *n* = 9. **c** The data suggest that peripheral B cells of tumor patients (*n* = 3) show significantly decreased Ca^2+^ influx after ADO treatment as compared to B cells of healthy donors (*n* = 9) analyzed with a two-way ANOVA; median with interquartile range. **d** The MFI of CD39 and CD73 on B cells was measured by FACS before and after treatment with ibrutinib or DMSO as a control. Ibrutinib induced a significant decrease in CD39 expression. Data were analyzed with a two-way ANOVA; median with interquartile range. **e** B cells were stimulated with CD40L, IL-4 and hemagglutinin and treated with ibrutinib for 6 days. Remaining ATP as measured by luminescence was significantly higher in ibrutinib-treated B cells as compared to control B cells (*n* = 7). Analyzed by two-way ANOVA; (*) *p* < 0.05; (**) *p* < 0.01; (***) *p* < 0.001; (****) *p* < 0.0001. **f** Adenosine concentrations measured by mass spectrometry for B cells of one donor stimulated for 6 days with CD40L, IL-4 and hemagglutinin and treated with ibrutinib. ATP was added at a concentration of 20uM

We observed a clear effect of ADO on BTK phosphorylation in B cells, which in turn is essential for the BCR-mediated Ca^2+^ flux. In order to further establish this coherency, isolated B cells were treated with ADO or with the BTK inhibitor ibrutinib overnight. Intracellular Ca^2+^ secretion was measured by FACS by the emission spectrum of Indo-1 after stimulation with anti-µ-F(ab’)_2_ (Fig. 3a). ADO as well as ibrutinib decreased the ability of B cells to secrete intracellular Ca^2+^, while ADO further enhanced the ibrutinib-mediated Ca^2+^ influx inhibition (*p* ≤ 0.01, Fig. 3b). Peripheral B cells of both healthy donors and cancer patients showed a decrease in Ca^2+^ influx after treatment with ADO (Fig. 3c). These results suggest that ADO inhibits Ca^2+^ influx by BTK inhibition in B cells of healthy donors and cancer patients.

### BTK is essential for the ADO production of B cells

Stimulated B cells were pre-treated with ibrutinib for six days. Next, the expression of the ectonucleotidases was measured by FACS and the hydrolysis of exogenous ATP was measured by luminescence. The expression of CD39 was significantly decreased in ibrutinib-treated B cells as compared to untreated controls (*p* ≤ 0.01, Fig. 3d). CD73 showed no differences after treatment with ibrutinib (*p* = 0.8). Additionally, the ability for hydrolysis of exogenous ATP was significantly reduced in ibrutinib-treated B cells as measured by luminescence assay. Experiments of seven representative donors are displayed in Fig. 3e (*p* ≤ 0.05). In order to confirm these results measured by luminescence, samples of one patient were additionally measured by mass spectrometry. Ibrutinib-treated B cells of this patient expressed less CD39 (MFI 55.7 vs. 31.8). Accordingly, these ibrutinib-treated B cells produced less ADO than the untreated control (Fig. 3f). These results suggest that BTK induces an upregulation of CD39 on B cells, which in turn increases the ability for ADO production.

### B cells in murine HNSCC express CD39 and CD73

**Fig. 4:**
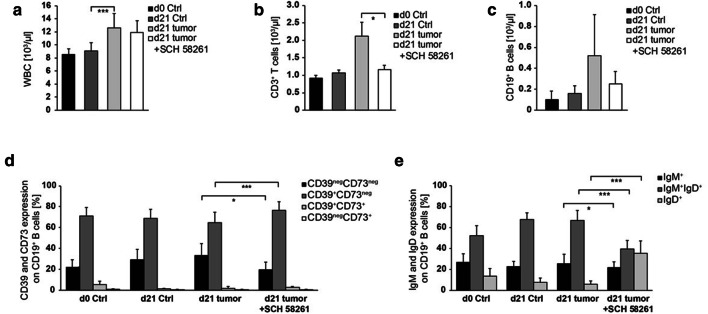
Cell populations in the peripheral blood of HNSCC-bearing mice. **a** Leukocytes were determined using animal blood counter (*n* = 57). **b–c** Populations of blood lymphocytes and B cells were analyzed by flow cytometry (*n* = 43/47). **d–e** The expression profile for CD39/CD73 and IgM/IgD was determined in peripheral B cells. Cell numbers per µl blood were calculated based on WBC. Blood of control and tumor-bearing mice, which were either left untreated or treated with the ADORA_2A_ antagonist SCH-58261, was analyzed at the tumor induction and after 21 days (*n* = 43/54)

**Fig. 5:**
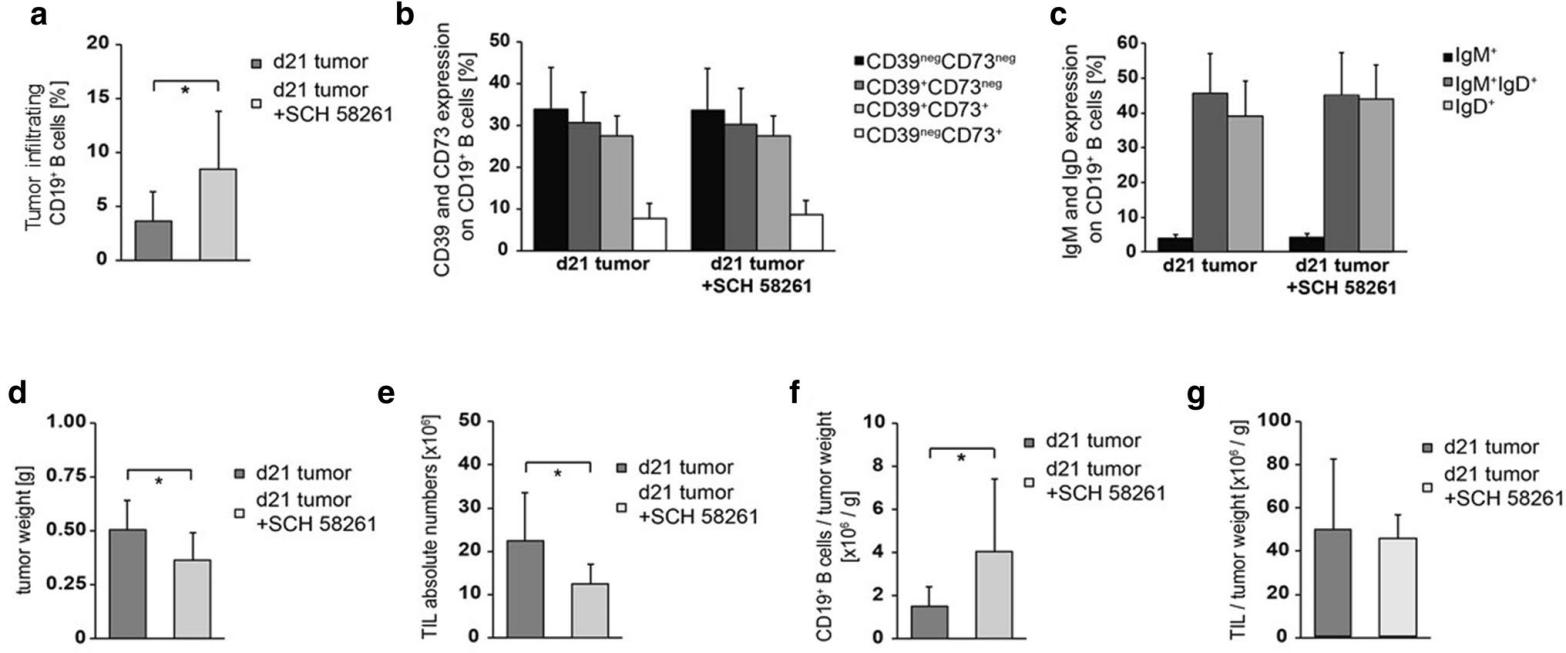
Tumor growth and TIL in HNSCC-bearing mice. HNSCC mice were either left untreated or treated with the ADORA_2A_ antagonist (SCH-58261). **a** At d21 after tumor induction, tumors were harvested for determination of CD19^+^ B cell frequency (*n* = 20). Infiltrating tumor cells were further characterized for **b** CD39 and CD73 expression (*n* = 22), as well as **c** IgM and IgD expression (*n* = 22). **d–e** Mice, which were treated with SCH-58261, showed a decrease in tumor weight and in the absolute number of tumor-infiltrating lymphocytes (*n* = 22/21). **f** However, the number of tumor-infiltrating B cells per g tumor was decreased (*n* = 21) and **g** the number of TIL per g tumor was stable (*n* = 21)

In order to prove the therapeutic potential of the ADO metabolism, we applied an orthotopic murine tumor model. In tumor-bearing mice, we observed a significant increase in white blood counts (WBC) 21 days after tumor induction, mainly caused by a strong increase in granulocytes and monocytes (Fig. 4a). Additionally, lymphocytes were significantly increased in the blood of tumor-bearing mice due to the increase in T and B cell numbers (Fig. 4b, c). However, the frequency of ADO-producing B_reg_ (CD39^+^CD73^+^) remained stable in tumor-bearing mice (Fig. 4d). Also, the percentages of IgM^+^, IgM^+^IgD^+^ or IgD^+^ B cells were not significantly changed between control mice and tumor-bearing mice (Fig. 4e). Tumor-infiltrating B cells (TIL-B) were detected by expression of CD19 (Fig. 5a). In contrast to peripheral blood, a greater proportion (25–30%) of these B cells expressed CD39 as well as CD73 simultaneously (Figs. 4d and 5b; d21 w/o treatment). TIL-B were more mature as compared to B cells found in the peripheral blood as based on IgD expression (Figs. 4e and 5c).

In our murine tumor model, we observed a high proportion of CD39^+^CD73^+^ TIL-B, which have the potential to produce ADO. We, therefore, assumed that increased concentrations of B cell-derived ADO contribute to an immunosuppressive microenvironment and tumor progression. To test this hypothesis, we applied the ADORA_2A_-inhibitor SCH-58261 in our tumor-bearing mice. ADORA_2A_ is expressed on murine lymphocytes, but not on tumor cells (Supplementary fig. S1b/c). After 21 days, the inhibition of ADORA_2A_ with SCH-58261 significantly reduced tumor growth as well as the absolute number of TIL (Fig. 5d, e). Yet, the number of TIL per tumor weight remained unchanged (Fig. 5f, g). Interestingly, the percentage of TIL-B increased significantly in treated mice (Fig. 5a), while their expression of CD39 / CD73 and their status of maturity (by IgM and IgD expression) remained unchanged (Fig. 5b, c). In contrast, the frequency of peripheral B cells was slightly reduced in treated mice (Fig. 4c), while the expression of CD39 and IgD was upregulated on peripheral B cells (Fig. 4d, e). Of note, the inhibition of ADORA_2A_ induced a significant decrease in the tumor mass even when the antagonistic therapy was started as late as one week after tumor initiation.

## Discussion

Immunosuppression is one of the major tumor escape mechanisms, and ADO is known to play an important role in the generation of an immunosuppressive tumor microenvironment [13, 14, 23–25]. Several cell types are capable of producing ADO, including regulatory immune cells, cancer cells as well as non-cellular vesicles, mainly due to the expression and activity of the ectonucleotidases, CD39 and CD73 on their surface [25–27]. Although the effect of ADO on T cell effector function has been extensively studied [13], the effect of ADO on B cells is still unknown. At the same time, it becomes evident that B cells may play an important role in the immune response of cancer patients [28]. Recently, we have shown that human peripheral B cells also express CD39 and CD73 on the cell surface and generate large amounts of exogenous ADO. Therefore, these B cells have regulatory characteristics, which enable them to suppress T cell functions, in vitro [12]. In fact, their ability to produce ADO is significantly higher than other immune cells, which are either CD39^+^CD73^neg^ (e.g., T_reg_, monocytes) or CD73^+^CD39^neg^ (e.g., T_Helper_ cells, T_Killer_ cells). However, we are well aware that adenosine production is only one functional aspect of tumor-infiltrating B cells. As more than 50% of B cells can produce adenosine, the same cells are probably also capable of secreting cytokines or to process antigens [29]. In view of this hypothesis, we support the idea that tumor-infiltrating B cells may serve as a positive prognostic marker in cancer patients [30, 31]. The correlation between humoral immune responses and different patterns of immune infiltrates in HNSCC patients has been described in detail by Lechner et al. [32]. While humoral immune responses were lower in HPV^+^ cancer patients, higher humoral immune responses were associated with CD4^+^ T cell-dominated immune infiltrates.

Our results demonstrate that the frequency of ADO-producing B_reg_ is lower in the TME as compared to the peripheral blood of cancer patients (Fig. 1a). We believe that the frequency is regulated by **(I)** the selective attraction of different B cell subsets into the TME as well as **(II)** the influence of the TME on stationary B cells. Nevertheless, even in the TME almost 50% of B cells are capable of producing extracellular ADO by the CD39/CD73 pathway.

Previously, we have demonstrated how chemotherapeutic drugs can influence the ADO production in B cells, in vitro and in vivo [17, 33]. We now describe the presence of ADO-producing B_reg_ in the tumor tissue of HNSCC in human and mice, in which the majority of B cells have a regulatory phenotype (CD39^+^CD73^+^). By their continuous production of ADO, B_reg_ help to create an immunosuppressive micromilieu, which is a characteristic feature of many solid tumors [13, 14]. Accordingly, immunosuppression by ADO is regularly regarded as a tumor promoting factor; and solid tumors contain high levels of endogenous immune-suppressive ADO as compared to the normal tissue [5, 13, 14]. In HNSCC patients, low levels of ADORA_2A_ correlate with low frequencies of T_reg_ and a high frequencies of CD8^+^ T cells, indicating a significantly improved survival rate [16].

The mechanism by which ADO suppresses T cells has been extensively analyzed and includes upregulation of cAMP after ADORA_2A_ activation [16, 24]. Consequently, T-cell proliferation and IFN-γ release are decreased [34]. In contrast to T cells, the knowledge regarding ADO signaling in B cells is very limited. Here, we demonstrate that extracellular ADO downregulates BCR-mediated signaling by decreased phosphorylation of BTK (Bruton’s tyrosine kinase). BTK is a central member of the intracellular signaling cascade after the activation of the common BCR. Mutations along the BTK gene *BTK* cause X-linked agammaglobulinemia (XLA), characterized by severe defects of the B-cell development and the innate immune system [35]. BTK phosphorylation induces downstream activation of Akt, NF-κB and Ca^2+^ influx [36, 37], which in turn regulates the activation of pro-inflammatory proteins [38]. Our previously published work has described the influence of ADO on the function of B cells [12]. This includes, e.g., reduced expression of cytokines (IL-6 and IL-8) and reduced proliferation of activated B cells in the presence of ADO.

In the past, other research groups have shown that extracellular ADO induces a reduction in Ca^2+^ influx in lymphocytes [39]. Our experiments now describe one of the underlying mechanisms. In detail, exogenous ADO decreases phosphorylation of BTK with a consequent decrease in Ca^2+^ influx in B cells of healthy donors and cancer patients, and this effect is dependent on the ADO receptor A2. In our experiments, the decrease in Ca^2+^ influx by ADO was further enhanced by the BTK inhibitor ibrutinib, indicating that either ADO or ibrutinib may utilize additional signaling events other than BTK to inhibit the calcium flux in B cells. The BTK inhibitor ibrutinib is well known as a treatment option in chronic lymphocytic leukemia and mantle cell lymphoma, in which ibrutinib silences the downstream pathways of ERK, PI_3_K, NF-κB and Akt, and induces apoptosis of malignant B cells [40, 41].

The therapeutic potential of ibrutinib in solid tumors is currently being evaluated by a series of research teams including our own group [42–44]. However, the prognostic benefit of these molecular changes in patients with solid tumors is still unknown.

**Fig. 6:**
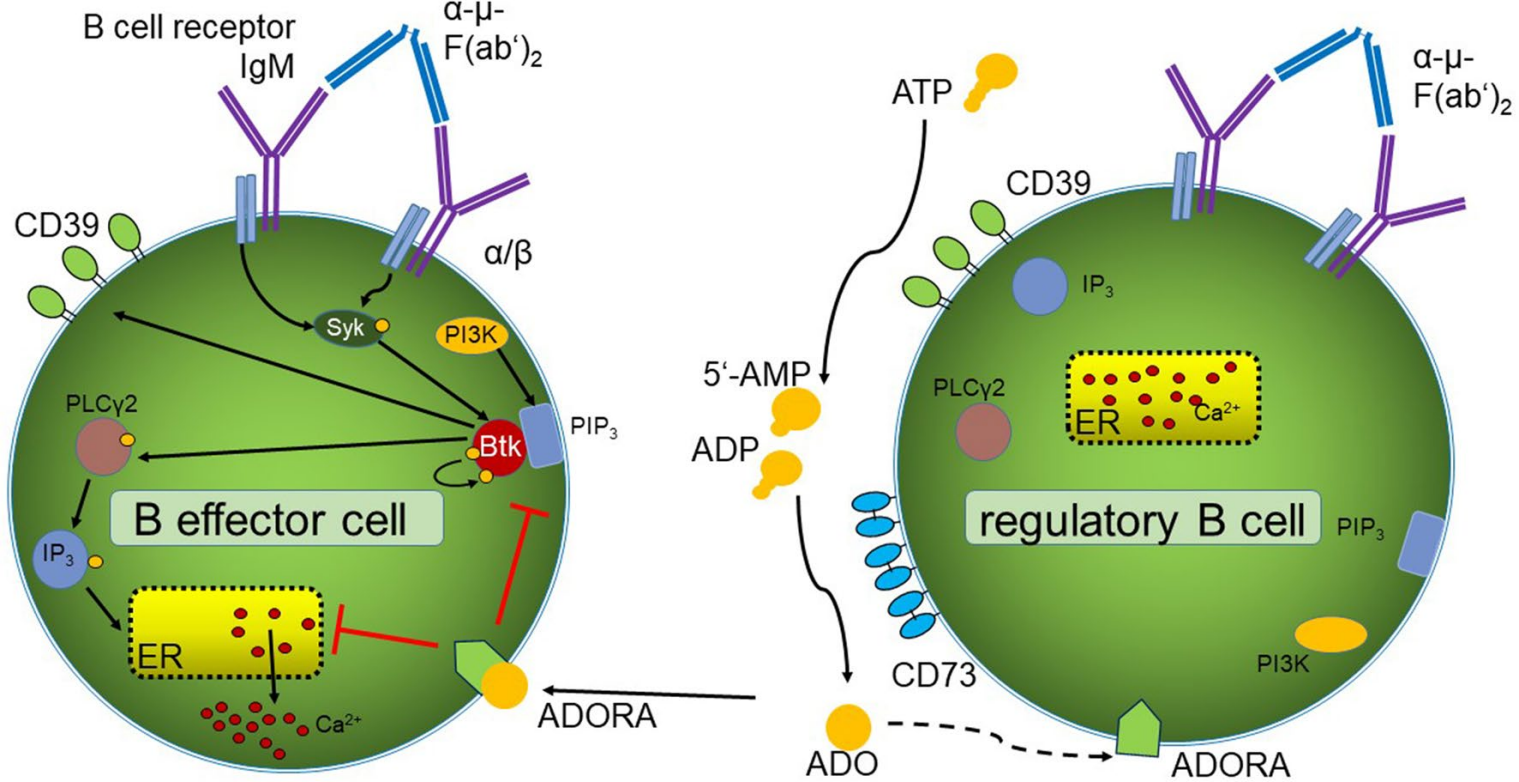
Adenosine affects the B cell receptor pathway. In B effector cells, binding of the antigen α-µ-F(ab’)_2_ to the BCR induces Syk and PIP3 activation supported by PI3K signaling. PIP3 recruits BTK, inducing auto-phosphorylation. The activated BTK activates PLCγ2 and IP3, binding to the endoplasmatic reticulum (ER), which secrets Ca^2+^. On B_reg_ cells, extracellular ADO is produced by hydrolysis of ATP by the ectonucleotidases CD39 and CD73. ADO binds to different ADO receptors, downregulating the auto-phosphorylation of BTK and the Ca^2+^ influx in CD73^neg^ B cells. In B_reg_ cells, no BTK phosphorylation was found upon binding of the antigen α-µ-F(ab’)_2_

In our experiments, BCR-induced BTK phosphorylation was detectable only in B_eff_, but not in B_reg_ (Fig. 2b). In contrast, only B_reg_ were able to produce ADO by co-expression of the ectonucleotidases CD39 and CD73. We, therefore, hypothesize that CD73^+^ B_reg_ are able to suppress BCR signaling in CD73^neg^ B_eff_ by ADO production in the tumor tissue as illustrated in Fig. 6. In addition, our results suggest a negative feedback mechanism in B cells, as ibrutinib decreases the production of extracellular ADO by downregulation of CD39 on B cells.

In knowledge of these molecular mechanisms, we hypothesize that blockade of the adenosine pathway may have a therapeutic potential. Others have previously shown that the inhibition of ADORA_2A_ in mice leads to a delayed growth of HNSCC tumors and enhances the anti-tumor response of CD8^+^ T cells [16]. Our own murine tumor model confirmed the idea that ADO signaling is a crucial factor contributing to tumor growth.

Other murine tumor studies have shown that the inhibition of ADORA_2A_ decreases the number of T cells in the tumor environment [13] and the metastasis of CD73^+^ tumors [23]. Our experiments now add to this knowledge by demonstrating that the number of tumor-infiltrating B cells increases during the inhibition of ADORA_2A_. At the same time, we observed an increased CD39^+^CD73^+^ co-expression, when murine tumor-infiltrating B cells were treated with the ADORA_2A_ inhibitor SCH-58261. The current literature describes a CD73^+^ B-cell subset, which is regularly found in the germinal centers [45]. Others have described that the expression of ectonucleotidases on B cells is dependent on their maturation status [46]. Also, the method of stimulation may have diverse effects on the maturation and expression of ADO-producing ectoenzymes on B cells, in vitro. As treated mice showed a significant decrease in tumor size in our experiments, it remains to be elucidated whether these cells are genuine B_reg_ or whether they belong to a specific mature B cell subset.

In the peripheral blood, the percentage of B_reg_ was low in healthy mice (~ 3%) as compared to healthy humans (~ 60%). However, in tumor-bearing mice, the B_reg_ frequency is increased in the peripheral blood as well as in tumor tissue. In contrast, the majority of human circulating B cells express CD39 and CD73 in healthy donors as well as in HNSCC patients. This observation could be explained by the fact that mice were kept under pathogen-free conditions, while the human immune system is constantly active due to continuous exposure to pathogenic agents. The function of ADO-producing B_reg_ may therefore be the suppression of excessive immune reaction in order to prevent tissue damage or autoimmunity [47].

## Conclusion

We demonstrate the presence of a novel ADO-producing B_reg_ population within the tumor microenvironment in mice and humans. A new model is proposed on how ADO-producing B_reg_ can influence the function of B_eff_ cells in healthy donors and cancer patients. Thus, the modulation of the ADO pathway in B cells may serve as a therapeutic approach for cancer patients.

## Electronic supplementary material

Below is the link to the electronic supplementary material.

Supplementary file1 (PDF 403 kb)

## Abbreviations

ADO: Adenosine
ADORA: Adenosine receptor A
BCR: B cell receptor
B_reg_: Regulatory B cells
BTK: Bruton’s tyrosine kinase
GAPDH: Glyceraldehyde 3-phosphate dehydrogenase
G6PD: Glucose-6-phosphate dehydrogenase
HNSCC: Head and neck squamous cell carcinoma
PBG-D: Porphobilinogen deaminase
RPL13A: Ribosomal protein L13a
SCC: Squamous cell carcinoma
TBP: TATA-box-binding protein
T_reg_: Regulatory T cells
