# Supplementary material for: Adenosine-producing regulatory B cells in head and neck cancer
Source: Cancer Immunol Immunother. 2020 Mar 7;69(7):1205–16. doi: 10.1007/s00262-020-02535-6 (PMC7303082; doi:10.1007/s00262-020-02535-6)
Supplement: Supplementary file 1 — Supplementary file1 (PDF 403 kb) [file 262_2020_2535_MOESM1_ESM.pdf]

Table S1. Clinicopathological parameters of the patients.

	Healthy Controls	Tumor patients (blood)	Tumor patients (TIL)
Mean age (range)	49.5 (20-84)	69.9 (47-90)	66.4 (47-90)
Sex			
Male	36	32	32
Female	24	10	7
T classification			
Tx		1	2
T1		8	5
T2		5	7
T3		14	14
T4		14	11
N classification			
N0		18	14
N1		6	7
N2		15	15
N3		3	3
M classification			
M0		37	36
M1		3	2
Mx		2	1
Relapse		5	3
Death		8	6
HPV-status			
Positive (p16)		2	5
Localization			
Pharynx		15	21
Larynx		13	8
Mouth		7	4
Nose		3	2
CUP		1	2
Other		3	2

Table S2. Genespecific primer and the specific UPL-Probe used for expression level measurements of ADOR at RNA level.

Gene	Primer left	Primer right	UPL-Probe No.
RPL13A	cccctgtttcaagggataaga	gaccatcaagcaccaggac	# 63
β-actin	attggcaatgagcggttc	cgtggatgccacaggact	# 11
PBG-D	tgccctggagaagaatgaag	cagcatcatgagggttttcc	# 79
G6PD	ctggtggccatggagaag	tgcatttcaacaccttgacct	# 22
GAPDH	gctctctgctcctcctgttc	acgaccaaatccgttgactc	# 60
TBP	gaacatcatggatcagaacaaca	atagggattccgggagtcat	# 87
ADORA1	gtcaagatccctctccggta	tcccaccacgaaggagag	# 66
ADORA2A	tgaccgctacattgccatc	tccaacctagcatgggagtc	# 3
ADORA2B	tctgtgtcccgctcaggta	gatgccaaaggcaaggac	# 56
ADORA3	aggatgtgcggtgcataaa	ccttagaaagggctcatcaca	# 11
Mouse ADORA1	ctgccagctttggtgtgac	accacaagggagagaatcca	#74
Mouse ADORA2A	tggtcctcacgcagagttc	ccgtcaccaagccattgta	#41
Mouse ADORA2B	gagctccatctttagcctcttg	tgtcccagtgaccaaacctt	#83
Mouse ADORA3	ggagctcgaaagttctctgg	tacttccccaggcaaacaga	#56
Mouse GAPDH	gggttcctataaatacggactgc	ccattttgtctacgggacga	#52
Mouse Beta actin	aaggccaaccgtgaaaagat	gtggtacgaccagaggcatac	#56

Figure S1.

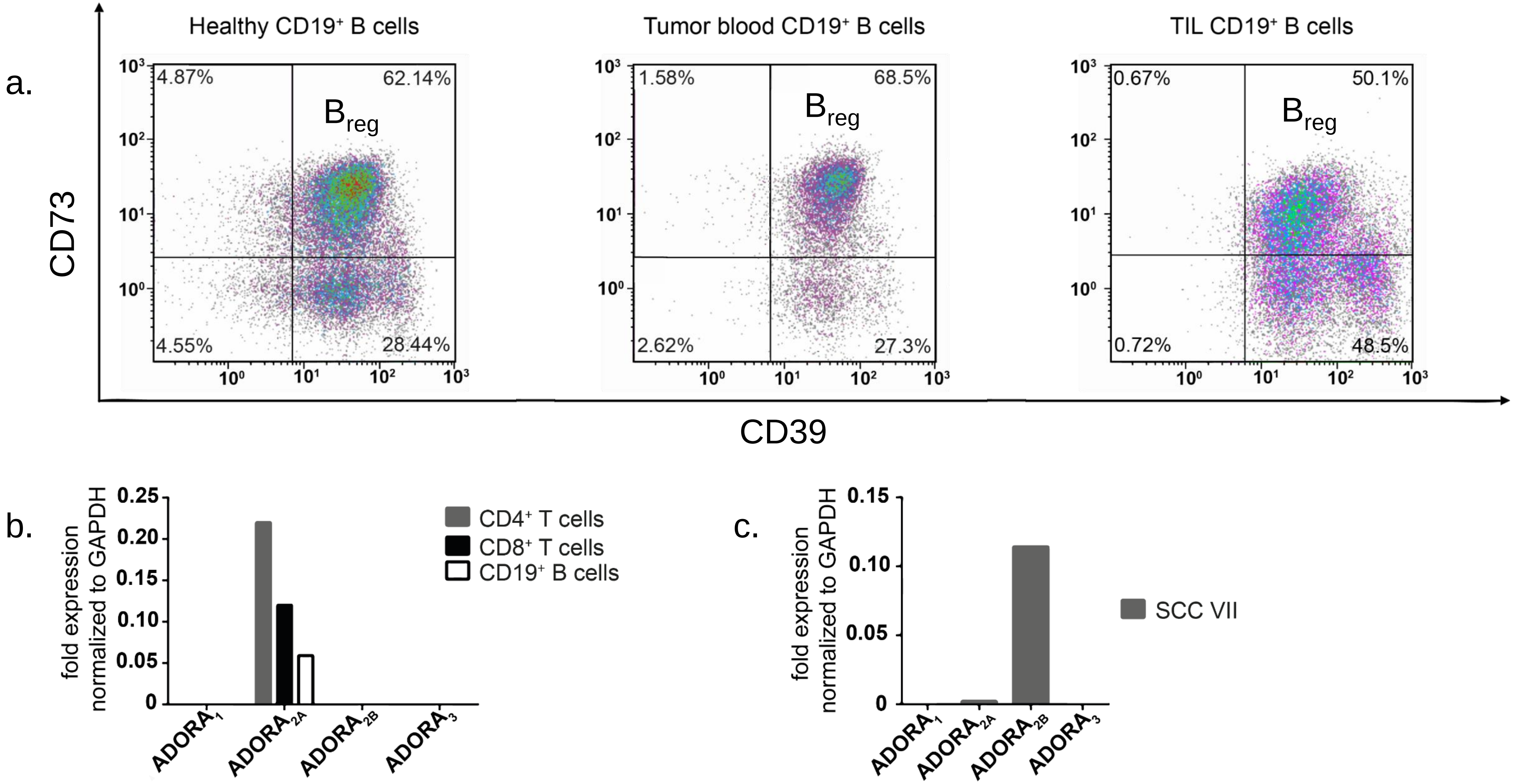

Fig. S1. (a) Characterization of human CD19+ B cells from healthy donors as compared to the phenotype of CD19+ B cells of the blood and the HNSCC tissue in exemplary plots measured by FACS. **(b/c)** Expression of ADO receptors on murine B cells and tumor cells.
